# Supplementary material for: Effects of Different Concentrations of Ganpu Tea on Fecal Microbiota and Short Chain Fatty Acids in Mice
Source: Nutrients. 2021 Oct 22;13(11):3715. doi: 10.3390/nu13113715 (PMC8618378; doi:10.3390/nu13113715)
Supplement: Supplementary file 1 [file nutrients-13-03715-s001.zip › nutrients-1306878-supplementary.pdf]

Supplemental Material of CW Manuscript

Table S1. Standard equations of eight SCFAs.

Name	Range (μg/mL)	Curve Equation	R^2	Limit of Quantitation
Acetic acid	0.2–400	y = 0.251995 * x + 0.004574	0.997	0.2
Propanoic acid	0.2–400	y = 0.350989 * x − 0.002259	0.998	0.2
Butanoic acid	0.2–400	y = 1.049535 * x + 1.913722E-004	0.998	0.2
Isobutyric acid	0.1–200	y = 0.936800 * x + 7.791846E-005	0.994	0.1
Valeric acid	0.1–200	y = 1.272853 * x − 1.945562E-004	0.997	0.1
Isovaleric acid	0.1–200	y = 1.493061 * x + 1.308246E-004	0.993	0.1
Hexanoic acid	0.1–200	y = 0.665629 * x + 0.001153	0.995	0.1
Isohexanoic acid	0.1–200	y = 0.467166 * x + 1.814266E-004	0.996	0.1

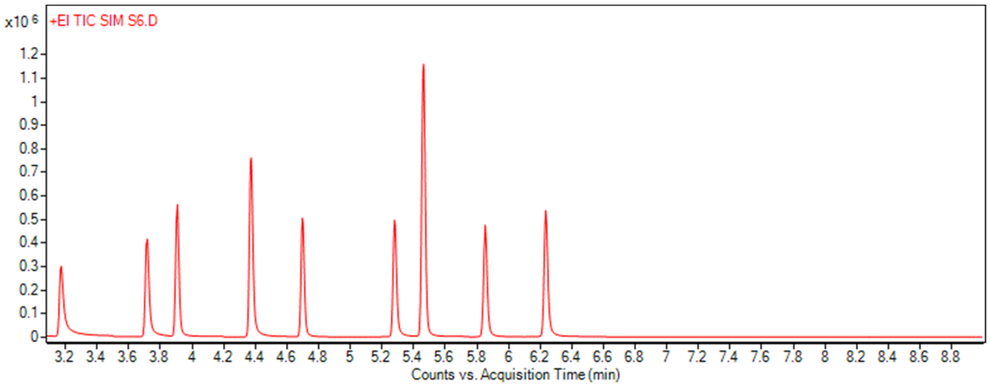

(**A**) standard solution

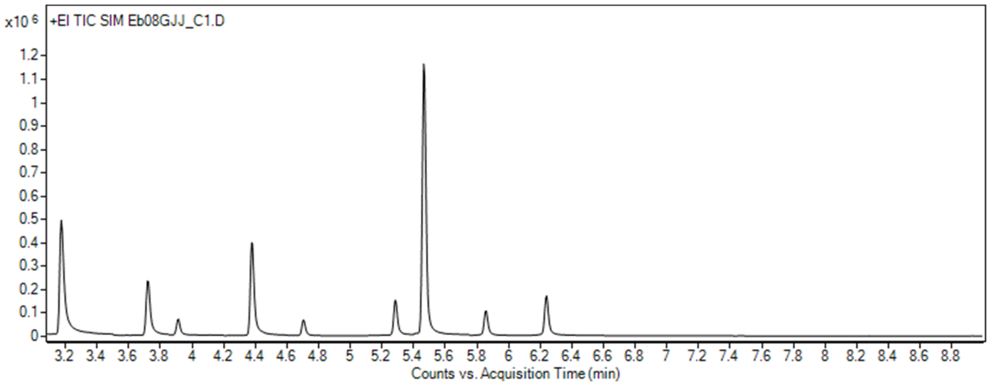

(**B**)Sample

Figure S1. the gas chromatographic diagram of the standard solution and sample.
